# Long-Chain and Very Long-Chain Ceramides Mediate Doxorubicin-Induced Toxicity and Fibrosis

**DOI:** 10.3390/ijms222111852

**Published:** 2021-11-01

**Authors:** Tom Kretzschmar, Mohamed M. Bekhite, Jasmine M. F. Wu, Daniela Haase, Martin Förster, Tina Müller, Sandor Nietzsche, Martin Westermann, Marcus Franz, Markus H. Gräler, P. Christian Schulze

**Affiliations:** 1 Department of Internal Medicine I, Division of Cardiology, University Hospital Jena, 07747 Jena, Germany; tom.kretzschmar2@med.uni-jena.de (T.K.); mohamed.el_saied@med.uni-jena.de (M.M.B.); jasmine.wu@med.uni-jena.de (J.M.F.W.); Daniela.Haase@med.uni-jena.de (D.H.); Martin.Foerster@med.uni-jena.de (M.F.); Marcus.Franz@med.uni-jena.de (M.F.); 2 Department of Anesthesiology and Intensive Care Medicine, University Hospital Jena, 07745 Jena, Germany; Tina.Mueller2@med.uni-jena.de (T.M.); markus.graeler@med.uni-jena.de (M.H.G.); 3 Center of Electron Microscopy, University Hospital Jena, 07743 Jena, Germany; Sandor.Nietzsche@med.uni-jena.de (S.N.); martin.westermann@med.uni-jena.de (M.W.)

**Keywords:** ceramides, reactive oxygen species, fibrosis, mitochondrial function, respiratory chain

## Abstract

Doxorubicin (Dox) is a chemotherapeutic agent with cardiotoxicity associated with profibrotic effects. Dox increases ceramide levels with pro-inflammatory effects, cell death, and fibrosis. The purpose of our study was to identify the underlying ceramide signaling pathways. We aimed to characterize the downstream effects on cell survival, metabolism, and fibrosis. Human fibroblasts (hFSF) were treated with 0.7 µM of Dox or transgenically overexpressed ceramide synthase 2 (FLAG-CerS2). Furthermore, cells were pre-treated with MitoTempo (MT) (2 h, 20 µM) or Fumonisin B1 (FuB) (4 h, 100 µM). Protein expression was measured by Western blot or immunofluorescence (IF). Ceramide levels were determined with mass spectroscopy (MS). Visualizations were conducted using laser scanning microscopy (LSM) or electron microscopy. Mitochondrial activity was measured using seahorse analysis. Dox and CerS2 overexpression increased CerS2 protein expression. Coherently, ceramides were elevated with the highest peak for C24:0. Ceramide- induced mitochondrial ROS production was reduced with MT or FuB preincubation. Mitochondrial homeostasis was reduced and accompanied by reduced ATP production. Our data show that the increase in pro-inflammatory ceramides is an essential contributor to Dox side-effects. The accumulation of ceramides resulted in a lipotoxic shift and subsequently mitochondrial structural and functional damage, which was partially reversible following inhibition of ceramide synthesis.

## 1. Introduction

Dox is a chemotherapeutic agent that belongs to the family of anthracyclines. It is used to treat various tumors. Due to its severe side-effects, especially cardiotoxicity, it is only used in limited situations [1]. Long-term use of Dox leads to dilated cardiomyopathy and fibrosis [2]. The underlying mechanisms of Dox toxicity are still not fully understood; in particular, its effects on cardiac fibrosis have not yet been thoroughly analyzed [3].

Ceramides are sphingolipids with a sphingosine backbone and attached fatty acids of various chain lengths [4] and they are involved in multiple essential cell signaling pathways [5]. Long chain ceramides have an attached fatty acid with a chain length of more than 14 C-Atoms (C14) and very- long chain ceramides with more than 22 C-Atoms (C22) and their accumulation are negatively associated with cell viability [6]. One possible way of ceramide generation is via ceramide synthases (CerS) and especially CerS2 as part of the salvage and sphingomyelin pathway [7,8,9]. The expression of CerS2 differs based on the origin of the individual cells and is mainly responsible for the formation of very long-chain ceramides [10] The accumulation of ceramides is associated with various diseases in different tissues, but its regulation and respective signaling pathways are not fully understood [6,11,12,13].

We hypothesized that the cytotoxic side-effects of Dox are linked to long-chain and very long-chain ceramide accumulation in fibroblasts of the failing myocardium. Thus, we focused on hFSF under treatment with Dox and studied the role of CerS2 in the underlying mechanisms concerning ceramide accumulation, mitochondrial viability, and apoptosis.

## 2. Results

### 2.1. Determination of Dox Concentration and Lethality Measurement Identified 0.7 µM Dox as Most Suited Concentration

Fibroblasts were incubated with 4 concentrations of Dox (0.01 µM, 0.25 µM, 0.4 µM and 0.7 µM) for 24 h. Subsequently, lethality measurement with Ethidium Homodimer- 2 (Eth-D2) (Supplementary Figure S1A) was performed to determine the most suited Dox concentration. Eth-D2 staining identified 0.7 µM Dox and was used for the subsequent experiments. Additionally, time-dependent lethality measurement was conducted with 0.7 µM Dox to confirm that a certain survival rate and recovery and proliferation were still observable (Supplementary Figure S1B). MT was used to assess the positive effects of the reduction in mitochondrial ROS on mitochondrial homeostasis. Concentration was adapted from Zhang et al. [14]. FuB was used to determine the beneficial effects of ceramide reduction in mitochondrial ROS production and homeostasis. Concentration was selected from Bouhet et al. [15]. Both concentrations were tested for lethality with Eth-D2, but no toxicity was detected (data not shown).

### 2.2. Dox Increases CerS2 Protein Expression and Ceramide Levels

**Figure 1:**
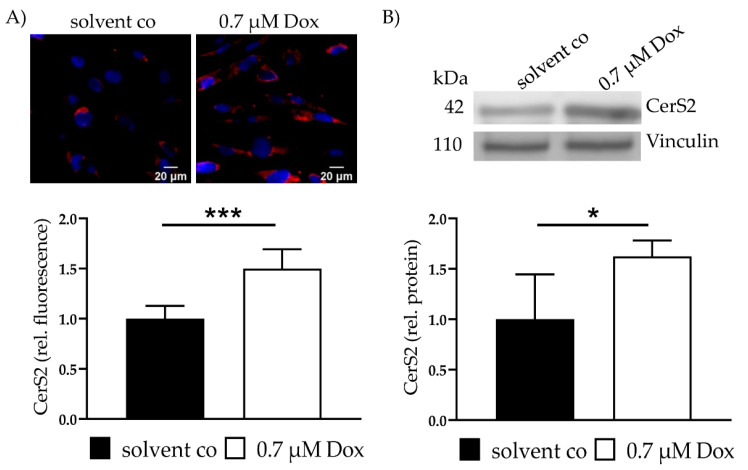
CerS2 and Ceramides—CerS2 protein expression and Ceramide level after Dox treatment and FuB preincubation. (**A**) CerS2 was stained with IF (red). Nucleus was stained blue. Intensity was determined with cLSM. Illustrated are two representative pictures. (**B**) CerS2 expression was assessed with WB with the same antibody. Illustrated is one representative blot. Depicted are mean values and standard deviation of minimum 3 biological replicates. Significances were calculated for all with a *t*-test (* *p* ≤ 0.05, ***p ≤ 0.001). solvent co = solvent control. Dox = Doxorubicin, FuB = Fumonisin B1.

CerS2 expression was assessed with IF and showed a 1.5 ± 0.19 (*p* < 0.001)-fold increase in CerS2 (Figure 1A). Increased CerS2 expression was also verified with WB (1.6 ± 0.16, *p* = 0.02) (Figure 1B). Increased levels of CerS4 and CerS5 were not observed. CerS6 showed a slight increase (data not shown).

Total ceramides were calculated by the addition of all measured ceramide species of each individual measurement (C14, C16, …, C24:1). Afterward, mean values and standard deviation were calculated and normalized to respective control.

MS revealed increased levels of total ceramides in Dox treated hFSF. Upon ceramide classification, MS revealed an increase in C14, C16, C18, C20, C22, C22:1, C24, and C24:1. FuB decreased the total ceramide level. Further analysis showed that the majority of ceramide species were reduced. C16 and C18 ceramides returned to normal level (Table 1).

### 2.3. Dox Damages Mitochondrial Structure by Increasing Mitochondrial ROS Levels

**Figure 2:**
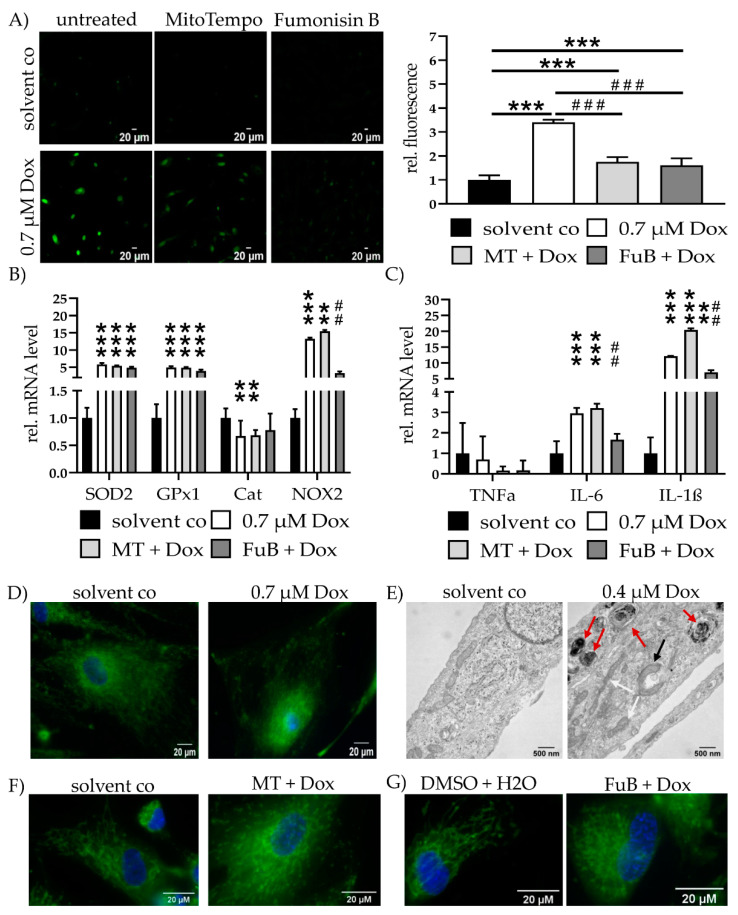
Mitochondrial Homeostasis—Mitochondrial ROS Production, Inflammation and Structure. (**A**) Mitochondrial ROS production was visualized with MitoSOX (green) and measured with cLSM. Pictures depict six representative images of three independent measurements. (**B**) mRNA expression levels of ROS scavenger (*SOD2, GPx1, Cat*) and inducer (*NOX2*). (**C**) mRNA expression levels of inflammatory cytokines (*TNFα, IL-6, IL-1β*). (**D**) Mitochondrial structure visualized with MitoTracker (green) and cLSM. Nucleus was stained blue. (**E**) Electron microscopy of cells treated with solvent co and 0.4 µM at 20,000× magnification. Red arrows indicate multilamellar bodies. Black arrow highlights an early autophagosome encapsulating a mitochondrion. White arrows show physiological tube-like mitochondria. (**F**) Cells were pre-treated with MT and subsequently treated with Dox. Mitochondrial structure visualized with MitoTracker (green) and cLSM. Nucleus was stained blue. Pre-treatment shows less impaired mitochondrial structure but no complete reversion to solvent co. (**G**) Cells were pre-treated with FuB and subsequently treated with Dox. Mitochondrial structure visualized with MitoTracker (green) and cLSM. Nucleus was stained blue. Pre-treatment with FuB restored some of the physiological net-like mitochondrial structure. Depicted are mean values and standard deviation of minimum 3 biological replicates. Significances were calculated with a *t*-test. (**/^##^ *p* ≤ 0.01, ***/^###^ *p* ≤ 0.001).

MitoSOX staining revealed a 3.4 ± 0.10 (*p* < 0.001)-fold increase in mitochondrial ROS. MT (1.7 ± 0.20, *p* < 0.001) or FuB (1.6 ± 0.29, *p* < 0.001) reduced mitochondrial ROS level. Additionally, mitochondrial ROS level were reduced compared to Dox (*p* < 0.001 for MT and FuB) (Figure 2A).

ROS homeostasis and inflammation status were further validated by the measurement of ROS-associated genes *SOD2*, *GPx1*, *Cat*, and *NOX2*, as well as cytokine mRNA expression of *TNFα IL-6* and *IL-1β* (Figure 2B,C). *SOD2* and *GPx1* were increased (5.6 ± 0.43, *p* < 0.001 for *SOD2*, 4.9 ± 0.34, *p* < 0.001 for *GPx1*). Both remained elevated after MT (5.4 ± 0.13, *p* < 0.001 for *SOD2*, 4.8 ± 0.19, *p* < 0.001 for *GPx1*) or FuB (4.8 ± 0.30, *p* < 0.001 for *SOD2*, 3.9 ± 0.38, *p* < 0.001 for *GPx1*). *Cat* was reduced in Dox (0.7 ± 0.28, *p* = 0.002) as well as MT (0.7 ± 0.10, *p* = 0.008). *Cat* expression level returned to a normal level after FuB (0.8 ± 0.30, *p* = 0.09). *NOX2* expression was increased (13.2 ± 0.35, *p* = 0.001) and remained unchanged with MT (15.4 ± 0.40, *p* = 0.007). FuB returned *NOX2* expression to normal level (3.3 ± 0.50, *p* = 0.053) and led to a significant reduction compared to Dox (*p* = 0.005). Dox and the inhibitors had no effect on TNFα expression. *IL-6* was elevated (3.0 ± 0.26, *p* < 0.001) and remained elevated with MT (3.2 ± 0.22, *p* < 0.001). FuB returned *IL-6* to normal level and a reduction compared Dox (*p* < 0.001). *IL-1β* was increased (12.1 ± 0.10, *p* < 0.001) and remained elevated under MT (20.4 ± 0.52, *p* < 0.001). FuB slightly reduced *IL-1β* (7.1 ± 0.66, *p* = 0.002) and was significantly reduced compared to Dox (*p* = 0.04) (Figure 2C).

MitoTracker staining showed an utterly ruptured structure, mitochondrial distribution, and only a rudimentary mitochondrial network (Figure 2D). Electron microscopy revealed beginning structural damage following 0.4 µM Dox treatment, which was characterized by an accumulation of multilamellar bodies (Figure 2E, red arrows). Simultaneously, physiological tube-like mitochondria were also identifiable in the same cell (Figure 2E, white arrows). An early autophagosome that encapsulated a mitochondrion targeted for digestion was detected (Figure 2E, black arrow) and 0.4 µM Dox was selected because the structural damage in 0.7 µM Dox was too severe.

MT, as well as FuB, restored in part the physiological tube and net-like structure of the mitochondria (Figure 2F,G). Both inhibitors were not completely able to restore the structure to normal level.

### 2.4. Dox impairs Mitochondrial Homeostasis and Function and Causes Apoptosis

Mitochondrial homeostasis was further assessed by the measurement of mitochondrial fusion–fission related gene expression (Table 2). *MFN1* was and remained reduced. *MFN2* was increased after Dox and did not change. *OPA1* was also reduced and was not reversible.

The fission gene *DRP1* was and remained reduced. *Mff* expression was not affected by Dox but was reduced under MT or FuB. Mitochondrial fission gene *FIS1* showed no expression changes after Dox but was reduced with MT or FuB.

Metabolism related gene expression was assessed to determine the effects of Dox induced mitochondrial impairment on cell metabolism. The focus was primarily on fatty acid metabolism, because of the suspected lipotoxic shift caused by increased ceramides. Fatty acid metabolism associated gene *DGAT1* was increased and did not change under MT or FuB. An elevated expression level was also detected for *DGAT2* and returned to normal level after pre-treatment with either MT or FuB. Expression of *ATGL* showed significantly increased level and remained comparatively high with MT. FuB slightly decreased *ATGL* expression. Dox led to a significant increase in *CD36*, which was unaffected by MT and was slightly decreased with FuB. Expression of the mitochondria membrane localized *CPT1B* was and remained reduced under any condition. *PDK4* was reduced under any condition. GDH remained unchanged (Table 3).

**Figure 3:**
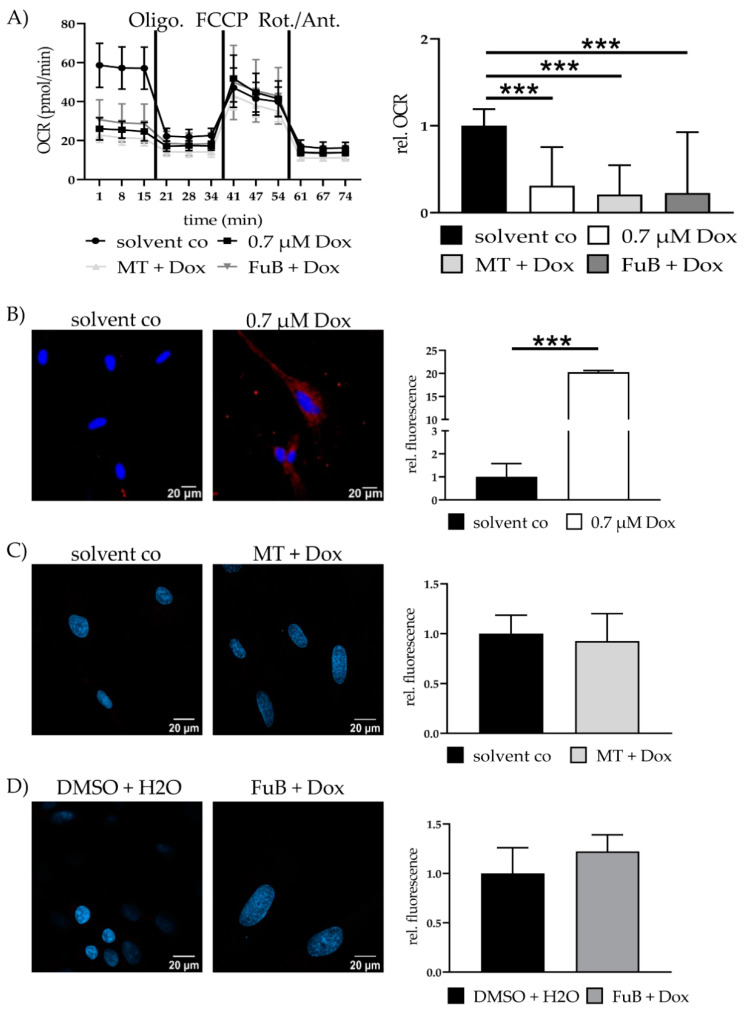
Mitochondrial activity and cell survival—Metabolic function and apoptosis. (**A**) Mitochondrial ATP production measured with Seahorse Analysis. Depictured is one representative measurement. (**B**) Cytochrome c delocalization from mitochondria to cytoplasm visualized with IF. Cytochrome c was stained red. Nucleus was stained blue. Shown are two representative images. (**C**) Cells were pre-treated with MT followed by Dox. Cytochrome c was stained red. Nucleus was stained blue Shown are two representative images. (**D**) Cells were pre-treated with FuB followed by Dox. Cytochrome c was stained red. Nucleus was stained blue. Shown are two representative images. Depicted are mean values and standard deviation of minimum three biological replicates. Significances were calculated with a *t*-test. (*** *p* ≤ 0.001).

Seahorse analysis revealed significantly reduced mitochondrial ATP production, characterized by a reduced Oxygen Consumption Rate (OCR) (0.3 ± 0.44, *p* < 0.001) (Figure 3A). The reduction did neither recover with MT (0.2 ± 0.34, *p* < 0.001) nor FuB (0.2 ± 0.70, *p* < 0.001).

**Figure 4:**
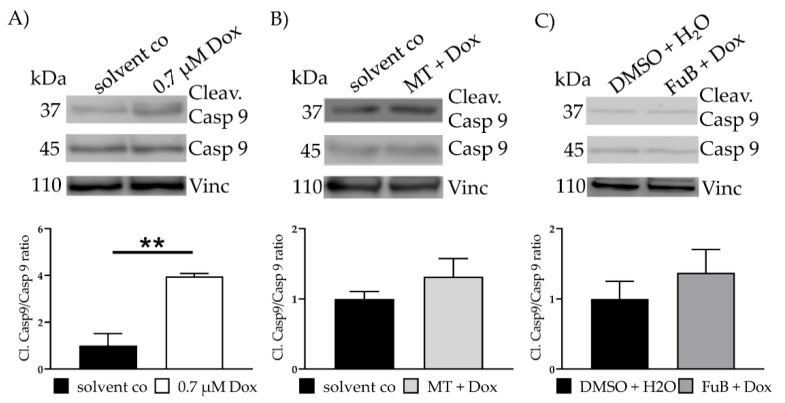
Apoptosis- Apoptosis induction measured with WB (**A**) Cells were treated with Dox for 24 h. Cleav.Casp9/Casp9 ratio was detected with WB. Shown is one representative blot. (**B**) Cells were pre-treated with MT followed by Dox for 24 h. Cleav.Casp9/Casp9 ratio was detected with WB. Shown is one representative blot. (**C**) Cells were pre-treated with FuB followed Dox for 24 h. Cleav.Casp9/Casp9 ratio was detected with WB. Shown is one representative blot. Depicted are mean values and standard deviation of minimum three biological replicates. Significances were calculated with a *t*-test. ** = significant to solvent co. (** *p* ≤ 0.01).

IF revealed translocation of cytochrome C from the mitochondria into the cytoplasm (20.3 ± 0.33, *p* < 0.001) (Figure 3B). MT and FuB pre-treatment returned cytochrome C intensity to normal level (Figure 3C,D). Increased cleaved-Casp9/Casp9- ratio (4.0 ± 0.12, *p* = 0.003) was detected with WB and returned to a normal level after MT (1.3 ± 0.26, *p* = 0.17) and FuB (1.4 ± 0.33, *p* = 0.37) (Figure 4A–C)

### 2.5. Dox Promotes Fibrosis

**Figure 5:**
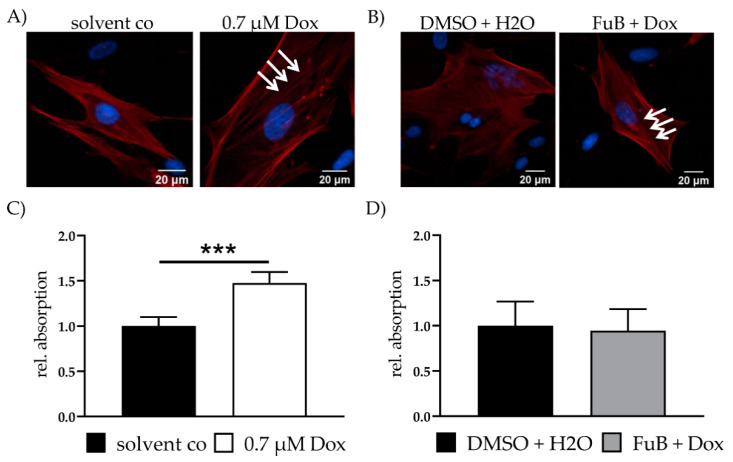
Fibrosis—ACTA2 expression and collagen production. (**A**) Fibrosis marker ACTA2 was visualized with IF. ACTA2 was stained red. Nucleus was stained blue. Dox treated cells show more defined ACTA2 filaments (white arrows). (**B**) Fibrosis marker ACTA2 was visualized with IF. ACTA2 was stained red. Nucleus was stained blue. FuB + Dox treated cells still show more defined ACTA2 filaments (white arrows). (**C**) Collagen in the cell supernatant was measured after Dox treatment, followed by 72 h growth medium incubation. (**D**) Collagen in the cell supernatant was measured after FuB followed by Dox treatment and additional 72 h growth medium. Depicted are the mean values and standard deviation of minimum three biological replicates. Significances were calculated with a *t*-test. (*** *p* ≤ 0.001).

Fibrosis is characterized by the disassembly of extracellular matrix proteins and replacement with fibrotic ones, e.g., collagen. The resulting “scar” impairs myocardial homeostasis and function. Because of this, fibrosis-associated genes were measured with qPCR. Dox showed a significant increase in all measured mRNA expressions related to the activation of fibrosis (Table 4). MT reduced *MMP14*, *TIMP2* and *TGF-β*, but still showed increased tendencies for all measured genes. Except for *TIMP1* and *ACTA2*, all of the measured genes returned to a normal expression level after FuB. Increased ACTA2 expression was further verified with IF. IF showed more defined α- smooth muscle actin (ACTA2) filaments in Dox treated cells (white arrows) compared to the diffused structure in the solvent co (Figure 5A). FuB pre-treatment had no effects on ACTA2 mRNA or protein expression (Figure 5B, Table 4).

Collagen was measured in the cell supernatant of Dox treated fibroblasts. Dox was replaced with a growth medium and the cells we incubated for additional 72 h. An increase in collagen was detected after this 72 h in Dox (1.48 ± 0.12, *p* < 0.001) (Figure 5C). FuB pre-treatment returned collagen to control level after 72 h incubation (0.95 ± 0.24, *p* = 0.71) (Figure 5D). We did not measure collagen in the cell supernatant and ACTA2 protein expression of MT preincubation because of the minor effects on the fibrosis related gene expression (Table 4).

### 2.6. CerS2 Knockdown Improves Dox-Mediated Mitochondrial Damage and Fibrosis

To further confirm the role of CerS2 in Dox-mediated cell toxicity, we knocked down CerS2 and subsequently treated the transfected cells with 0.7 µM Dox. Transfection success was assessed with WB (0.45 ± 0.33, *p* = 0.03) (Figure S2A). FACS was performed, to confirm the functional effects (Figure S2B). FACS measurement showed increased ceramide level in siCerS2 + 0.7 µM Dox compared to siRNA control + solvent co. The positive control (siRNA control + 0.7 µM Dox) showed further elevated ceramide levels compared to the other two conditions.

**Figure 6:**
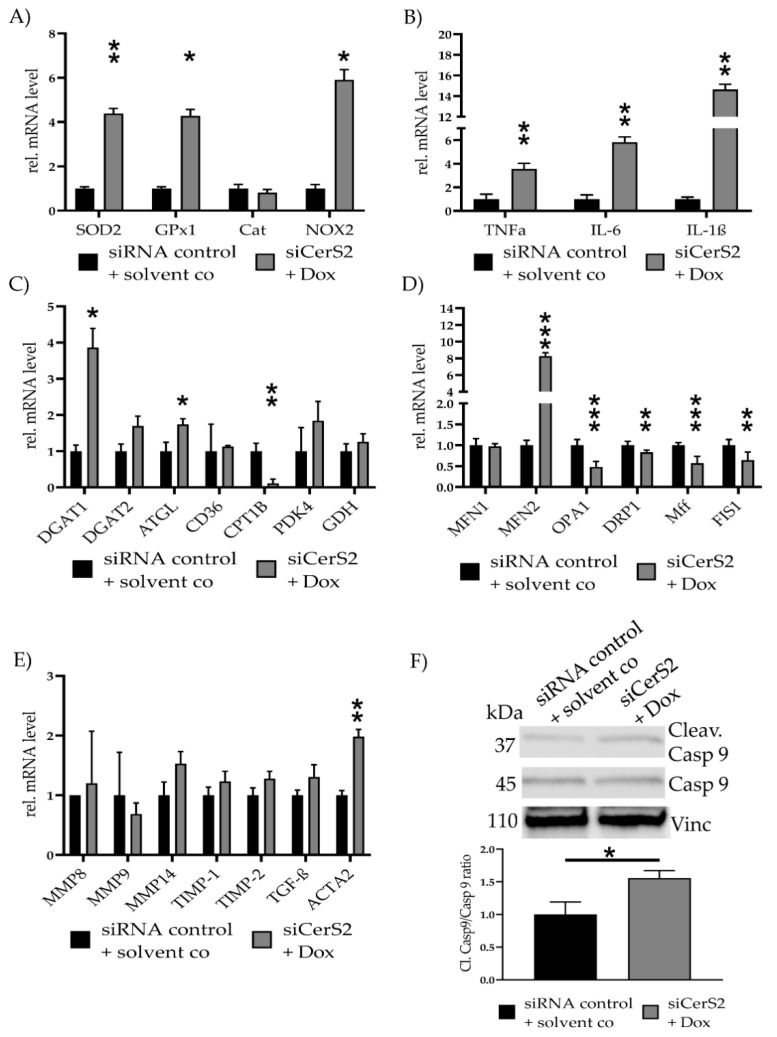
siCerS2 + Dox—Various effects of CerS2 kd determined with qPCR. hFSF were transfected with siRNA control or siCerS2 followed by 24 h incubation of solvent co or Dox. mRNA expressions were assessed with qPCR. (**A**) ROS scavenger (*SOD2, GPx1, Cat*) and inducer (*NOX2*) expression. (**B**) Inflammatory cytokine expression of *TNFα, IL-6*, and *IL-1β*. (**C**) Metabolism associated gene expression of fatty acid metabolism (*DGAT1, DGAT2, ATGL, CD36, CPT1B*), pyruvate metabolism (*PDK4*), and glutamate metabolism (*GDH*). (**D**) Mitochondria fusion (*MFN1, MFN2, OPA1*) and fission (*DRP1, Mff, FIS1*) related mRNA expression. (**E**) Fibrosis associated mRNA expression. (**F**) Cleav.Casp9/Casp9 ratio was detected with WB. Shown is one representative blot. Depicted are mean values and standard deviation of minimum three biological replicates. Significances were calculated with a *t*-test. * = significant to siRNA control + solvent co (* *p* ≤ 0.05, ** *p* ≤ 0.01, *** *p* ≤ 0.001).

We repeated all qPCRs to evaluate the effects of partial CerS2 reduction in the observed effects of Dox induced cell toxicity (Figure 6). SOD2 (4.38 ± 0.23, *p* = 0.009), GPx1 (4.28 ± 0.29, *p* = 0.02) and NOX2 (5.90 ± 0.47, *p* = 0.05) were increased in siCerS2 + Dox (Figure 6A). Inflammatory cytokine expression showed increased level for TNFα (3.55 ± 0.48, *p* = 0.008), IL-6 (5.82 ± 0.44, *p* = 0.002) and IL-1β (14.63 ± 0.51, *p* = 0.004) (Figure 6B). Assessment of metabolic gene expression revealed an increase for DGAT1 (3.86 ± 0.53, *p*= 0.02) and ATGL (1.74 ± 0.16, *p* = 0.05) and a decrease for CPT1B (0.11 ± 0.12, *p* = 0.004) (Figure 6C).

Mitochondrial fusion and fission-related gene expression levels were measured again (Figure 6D). The qPCR revealed a significant reduction of *OPA1* (0.46 ± 0.13, *p* < 0.001), *DRP1* (0.83 ± 0.04, *p* = 0.01), *Mff* (0.55 ± 0.18, *p* < 0.001), and *FIS1* (0.64 ± 0.21, *p* = 0.008). *MFN2* (7.12 ± 0.30, *p* < 0.001) expression increased, while *MFN1* showed no alteration.

Fibrosis related gene expression showed increased tendencies, but none were significantly altered, except for *ACTA2*. *ACTA2* was increased for siCerS2 + Dox (1.98 ± 0.12, *p* = 0.005) (Figure 6E). Cleav. Casp9/Casp9 ratio was still increased in siCerS2 + Dox (1.56 ± 0.11, *p* = 0.02) but the ratio was lower if directly compared to only Dox (Figure 6F).

### 2.7. CerS2 Overexpression Leads to an Increased Ceramide Production

**Figure 7:**
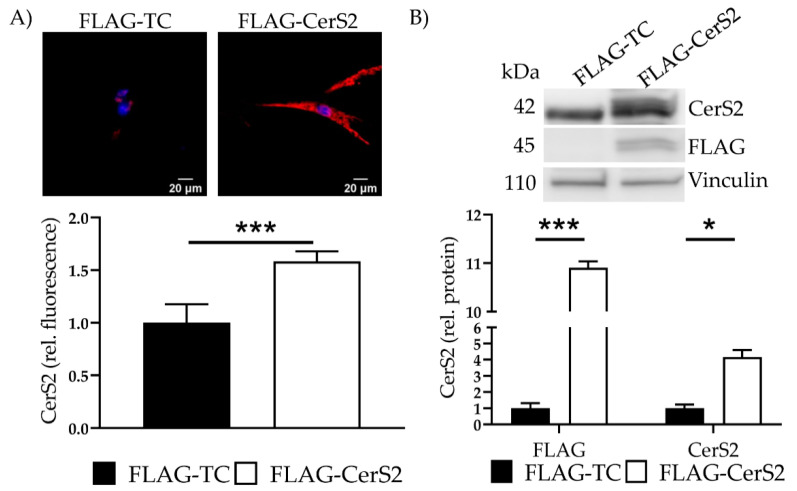
FLAG-CerS2—CerS2 protein expression and ceramide levels. (**A**) CerS2 was stained (red). Nucleus was stained blue. Intensity was determined with cLSM. Illustrated are two representative pictures. (**B**) Marker protein FLAG and CerS2 expression were assessed with WB. Shown is one representative blot. Depicted are mean values and standard deviation of minimum three biological replicates. Significances were calculated with a *t*-test. * = significant to FLAG-TC (* *p* ≤ 0.05, *** *p* ≤ 0.001).

We confirmed overexpression with IF and determined a 1.6 ± 0.1 (*p* < 0.001) increase (Figure 7A). Additionally, transfection showed a 10.9 ± 0.13-fold (*p* < 0.001) increase in the marker protein FLAG and a 4.2 ± 0.44 fold (*p* = 0.04) increase in CerS2 (Figure 7B). Changes of CerS5 and CerS6 were not observed (data not shown).

MS revealed increased total ceramide levels as well as very long chain ceramides. Moreover, a significant increase in C16, C18, C22, C24 and C24:1 was detected (Table 5).

FuB reduced ceramide level in FLAG-CerS2. C14 was significantly reduced with FuB while other ceramides returned to normal level (Table 6).

### 2.8. CerS2 Overexpression Impairs Mitochondrial Structure and Function

**Figure 8:**
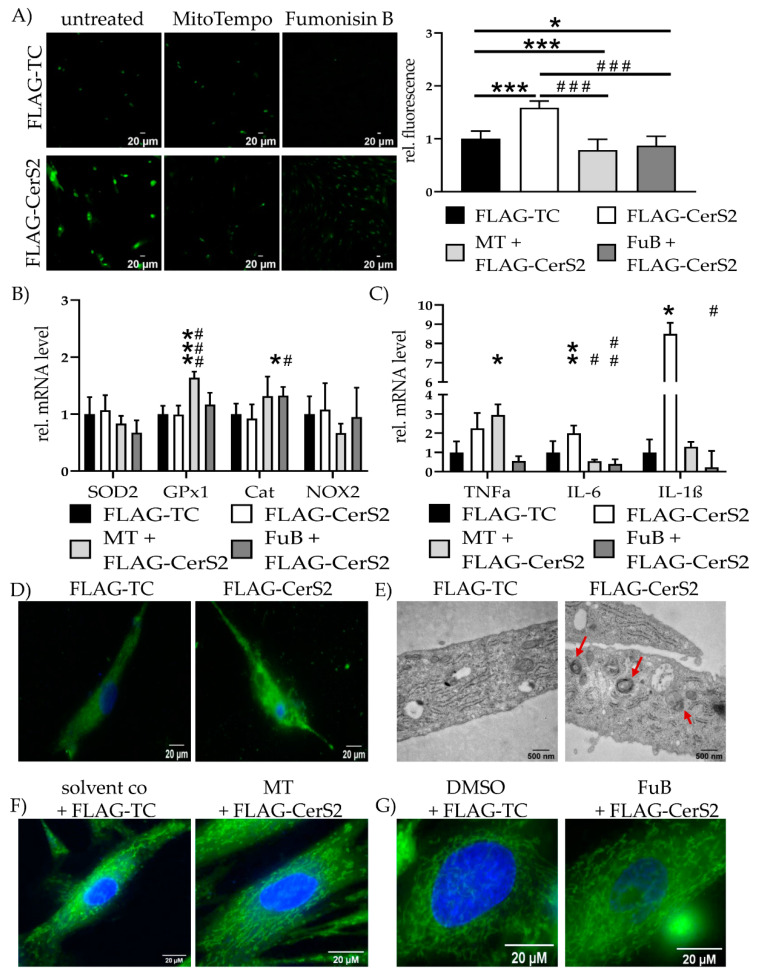
Mitochondrial Homeostasis—Inflammation and structure in FLAG-CerS2. (**A**) Mitochondrial ROS production was visualized with MitoSOX (green) and measured with cLSM. Pictures depict six representative images of three independent measurements. (**B**) mRNA expression levels of ROS scavenger (*SOD2, GPx1, Cat*) and inducer (*NOX2*). (**C**) mRNA expression levels of inflammatory cytokines (*TNFα, IL-6, IL-1β*). (**D**) Mitochondrial structure visualized with MitoTracker (green) and cLSM. Nucleus was stained blue. (**E**) Electron microscopies of cells of the FLAG-TC or FLAG-CerS2 condition at 20,000× magnification. Red arrows indicate multilamellar bodies. (**F**) Cells were pre-treated with MT and subsequently transfected. Mitochondrial structure visualized with MitoTracker (green) and cLSM. Nucleus was stained blue. Pre-treatment shows improved mitochondrial structure. (**G**) Cells were pre-treated with FuB and subsequently transfected. Mitochondrial structure visualized with MitoTracker (green) and cLSM. Nucleus was stained blue. Pre-treatment with FuB restored some of the physiological net-like mitochondrial structure. Depicted are mean values and standard deviation of minimum three biological replicates. Significances were calculated with a *t*-test. * = significant to FLAG-TC. # = significant to FLAG-CerS2. (*/# *p* ≤ 0.05, **/## *p* ≤ 0.01, ***/### *p* ≤ 0.001).

Increased CerS2 expression and ceramide production increased mitochondrial ROS production (1.6 ± 0.12, *p* < 0.001) and was reduced with MT (0.79 ± 0.20, *p* < 0.001) and FuB (0.87 ± 0.18, *p* = 0.02) (Figure 8A). Both reduced mitochondrial ROS production compared to FLAG-CerS2 (*p* < 0.001 for MT, *p* < 0.001 for FuB). Preincubation with MT increased *GPx1* (1.6 ± 0.11) expression compared to FLAG-TC (*p* < 0.001), as well as FLAG-CerS2 (*p* < 0.001) (Figure 8B). FuB increased *Cat* expression (1.3 ± 0.15) compared to FLAG-TC (*p* = 0.05) and FLAG-CerS2 (*p* = 0.04).

FLAG-CerS2 showed increased *TNFα* tendency but was only significant with MT (2.9 ± 0.55, *p* = 0.04). *IL-6* was increased in FLAG-CerS2 (2.0 ± 0.4, *p* = 0.03) and returned to normal level after MT (0.5 ± 0.08, *p* = 0.26) or FuB (0.4 ± 0.24, *p* = 0.10). Both were significantly decreased compared to FLAG-CerS2 (*p* = 0.02 for MT, *p* = 0.006 for FuB). *IL-1β* (8.5 ± 0.57, *p* = 0.05) expression was increased and returned after MT (1.3 ± 0.25, *p* = 0.58). FuB decreased *IL-1β* (0.2 ± 0.85) to normal level and significant compared to FLAG-CerS2 (*p* = 0.03) (Figure 8C).

Qualitative MitoTracker staining displayed severely impaired mitochondrial integrity and structure (Figure 8D). The structural impairment was also confirmed with electron microscopy. FLAG-CerS2 showed multilamellar bodies (Figure 8E, red arrows), indicating late lysosomes and autophagy. Pre-treatment with MT or FuB restored in part the physiological net-like structure of the mitochondria (Figure 8D,F). The beneficial effects of FuB seem to be more prominent than the positive effects of MT.

### 2.9. FLAG-CerS2 Leads to Reduced Mitochondrial Structure and Function and Induces Apoptosis

Mitochondria fusion gene *MFN1* was significantly reduced. MT returned *MFN1* expression to control level, while FuB significantly reduced *MFN1* level compared to FLAG-TC and FLAG-CerS2. *MFN2* expression was reduced and returned after MT and FuB. Preincubation with both inhibitors reduced *OPA1* expression. FuB reduced *DRP1* expression. *Mff* was reduced in FLAG-CerS2 and remained reduced with FuB. MT returned *Mff* expression to normal level. *FIS1* showed no expression changes but was reduced with MT or FuB. *FIS1* was reduced compared to FLAG-CerS2 under FuB (Table 7).

**Figure 9:**
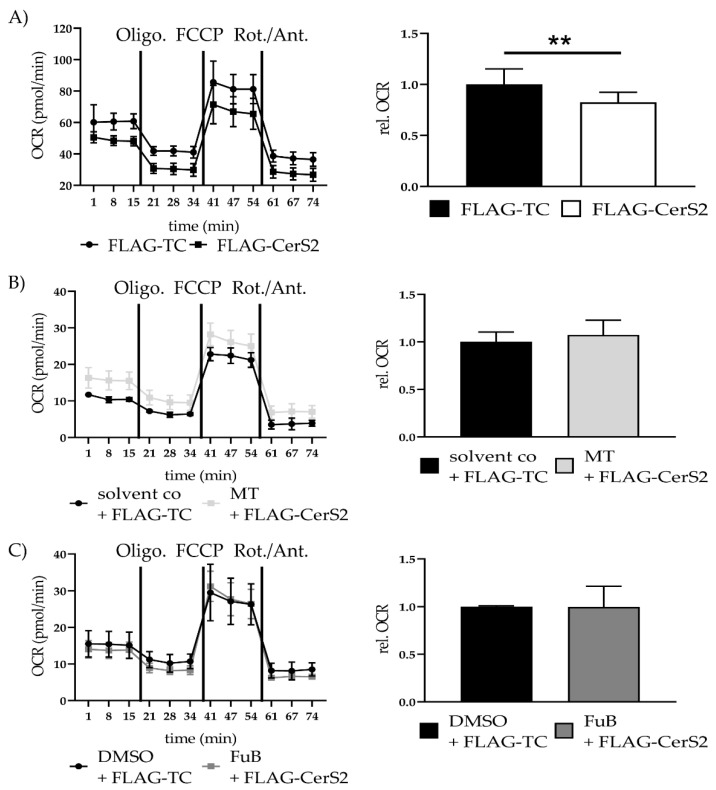
Mitochondrial Homeostasis—Metabolic function in FLAG-CerS2 determined with Seahorse Analysis. (**A**) Mitochondrial ATP production measured with Seahorse Analysis. Shown is one representative measurement. (**B**) Mitochondrial ATP production measured with Seahorse Analysis of FLAG-CerS2 hFSF pre-treated with MT. Shown is one representative measurement (**C**) Mitochondrial ATP production measured with Seahorse Analysis of FLAG-CerS2 hFSF pre-treated with FuB. Shown is one representative measurement. Depicted are mean values and standard deviation of minimum three biological replicates. Significances were calculated with a *t*-test. ** = significant to FLAG-TC. (** *p* ≤ 0.01).

Seahorse analysis showed a significant reduction in mitochondrial ATP production (0.82 ± 0.10, *p* = 0.003). MT (1.07 ± 0.16, *p* = 0.63) and FuB (1.0 ± 0.22, *p* = 0.98) returned ATP production to normal level (Figure 9A–C).

*DGAT1* expression was not altered. FuB reduced *DGAT1* compared to FLAG-CerS2. *DGAT2* expression was and remained reduced with MT and FuB. *CD36* expression was increased. FuB pre-treatment returned *CD36* to normal level and was significantly reduced compared to FLAG-CerS2. *CPT1B* expression was reduced. *PDK4* and *GDH* were unaltered (Table 8).

**Figure 10:**
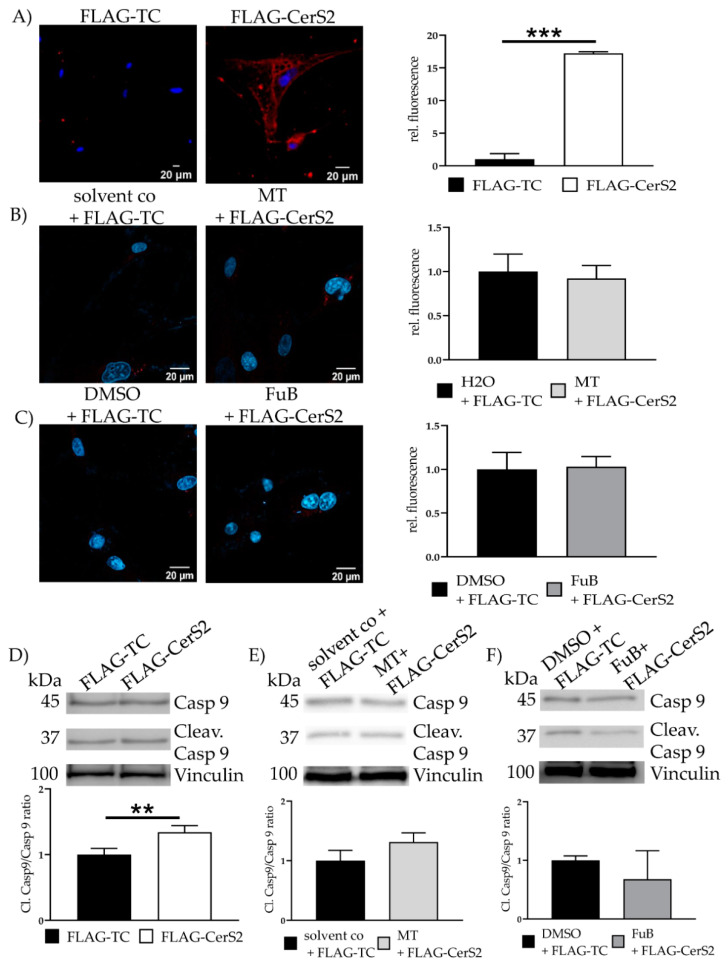
Apoptosis Initiation—Cytochrome C and Cleav. Casp 9 protein level in FLAG- CerS2. (**A**) Cytochrome c delocalization from mitochondria to cytoplasm visualized with IF. Cytochrome c was stained with red. Nucleus was stained blue. Shown are two representative images (**B**) Cells were pre-treated with MT and transfected afterwards. Cytochrome c delocalization from mitochondria to cytoplasm visualized with IF. Cytochrome c was stained with red. Nucleus was stained blue. Shown are two representative images (**C**) Cells were pre-treated with FuB and transfected afterwards. Cytochrome c delocalization from mitochondria to cytoplasm visualized with IF. Cytochrome c was stained with red. Nucleus was stained blue. Shown are two representative images. (**D**) WB analysis of cleaved caspase 9/caspase 9 ratio in FLAG-CerS2. Shown is one representative blot. (**E**) WB analysis of MT pre-treated FLAG-CerS2. Depicted is one representative blot. (**F**) WB analysis of FuB pre-treated FLAG-CerS2. Depicted is one representative blot. Depicted are the mean values and standard deviation of minimum three biological replicates. Significances were calculated with a *t*-test. (** *p* ≤ 0.01, *** *p* ≤ 0.001).

Cytochrome C was detectable in the cytosol of FLAG-CerS2 (17.2 ± 0.24, *p* < 0.001) (Figure 10A). MT and FuB preincubation reduced Cytochrome C to a normal level (Figure 10B, C). Increased cleaved caspase 9/caspase 9- ratio was detected (1.34 ± 0.10, *p* = 0.01) and returned to baseline level after MT (1.3 ± 0.16, *p* = 0.09) and FuB (0.68 ± 0.49, *p* = 0.25) (Figure 10C–E).

FLAG-CerS2 led to increased *MMP8* expression and showed increased tendencies for *MMP9* and *TGF-ß*. Under FuB *MMP8* and *MMP9* expression were no longer detectable (Table 9).

## 3. Discussion

Despite severe side-effects that include cardiotoxicity, Dox remains one of the most established and effective chemotherapeutic agents [1]. Nevertheless, the specific mechanisms of Dox-mediated cardiotoxicity and myocardial fibrosis remain to be elucidated [16,17]. Here, we have shown that Dox treatment of fibroblasts results in an increase in ceramides, also observable in CerS2 overexpression. This ceramide accumulation led to inflammation, as characterized by increased cytokines and mitochondrial ROS levels. This resulted in mitochondrial damage promoting apoptosis, all consistent with a lipotoxic phenotype. The detrimental effect on mitochondria is improved after reducing mitochondrial ROS levels and reducing ceramide levels. Additionally, the knockdown of CerS2 through gene silencing showed comparable results to FuB incubation with respect to mitochondrial homeostasis. Therefore, the current data link the cytotoxic properties of Dox to the accumulation of pro-inflammatory and lipotoxic ceramide species. Furthermore, mitigation of ceramides is beneficial for fibrosis reduction. Our findings highlight the role of long-chain and very long-chain ceramides, specifically produced by CerS2, to Dox associated adverse effects in fibroblasts.

The increase in ceramide species resulted in a lipotoxic phenotype. This is supported by the upregulation of the respective fatty acid associated genes *DGAT1* [18], *DGAT2* [19], *ATGL* [20] and *CD36* [21] and downregulation of *CPT1B* [22]. The lipotoxic shift, in turn, instigated increased mitochondrial ROS production followed by mitochondrial damage and the promotion of apoptosis. The observed increase in mitochondrial ROS production and cytokine mRNA levels is characteristic of inflammation and cellular damage [23]. Interestingly, even though ceramide levels were elevated in FLAG-CerS2, mitochondrial ROS levels and cytokine mRNA expression were higher in Dox-treated cells. These findings indicate a contributing role of ceramide species in Dox-mediated cytotoxicity and additional mechanisms involved in the pro-inflammatory activation. This is also supported by the reducing effects of FuB. FuB is an unspecific CerS inhibitor and returned all previously increased ceramide species equally in Dox and FLAG-CerS2. Additionally, in FLAG-CerS2 FuB significantly reduced C14 ceramides. CerS2 has no affinity for C14 ceramides [24] which was observable by the unchanged C14 level in FLAG-CerS2. That hints, that the detected reduction in C14 by FuB could be linked to CerS other than CerS2. The reduction in ceramides is characterized by reduced *IL-6*, *IL-1β*, mitochondrial ROS production, and recovered mitochondrial homeostasis.

MT had no effects on cytokine mRNA levels in Dox-treated cells, while it increased TNFα expression and reduced IL-6, IL-1β level in FLAG-CerS2, even though mitochondrial ROS levels were reduced in both. The increase in TNFα could be explained the varying transfection efficiency. This is further supported by the increased tendency of TNFα in only FLAG-CerS2 cells. The reduction of IL-1β and IL-6 by FuB shows that the reduction in mitochondrial ROS can be beneficial for the inflammatory status if there is a singular cause, such as specifically increased long chain and very long chain ceramides. The siCerS2 data support these findings due to the same expression patterns such as Dox-treated hFSF. Here, also, all cytokines were increased and showed no improvement by the reduction in CerS2, followed by Dox incubation.

This further indicates that the effects of Dox on inflammation are multiple and too profound to be reduced by the inhibition of mitochondrial ROS or ceramides alone.

There was no increase in the TNFα expression in Dox treated cells. Three out of 11 experiments showed very high values for solvent co, highly increasing standard deviation and preventing the detection of the expected increase.

Interestingly, neither the preincubation with MT or FuB reduced mRNA level of the ROS-Scavengers SOD2 or GPx1, indicating that mitochondrial ROS level or ceramides are not involved in the expression regulation. This is further indicated by the respective FLAG-CerS2 data, which showed also no effects on SOD2 and GPx1. Only Catalase was positively affected by FuB preincubation implying a correlation between ceramides and this specific ROS scavenger. Dox led to an increase in the ROS inducer NOX2, which was not affected by MT. Our data concur with McLaughlin et al. who also identified increased NOX2 level after DOX treatment [25]. The reduction in mitochondrial ROS level with MT had no beneficial effects on NOX2 expression, indicating that mitochondrial ROS production does not affect this specific cytosolic ROS inducer. FuB reduced NOX2 expression when compared to Dox-treated cells, but still showed an increased tendency. This shows the beneficial effects of ceramide reduction not only on mitochondrial, but also cytosolic ROS production. This is further supported by the siCerS2 data. Here also, SOD2 Gpx1 were increased while *Cat* returned to a normal level comparable to the Dox data. NOX2 was also increased but showed comparable level to FuB + Dox. Mitochondrial fusion and the resulting broad net-like structures are essential for mitochondrial function and cellular viability [26]. Increased ceramide levels cause structural impairment of mitochondria net-like structure. Electron microscopy showed a pathological accumulation of multilamellar bodies in Dox-treated and transfected cells, indicating mitochondrial digestion and autophagy in both cases. These results concur with Law et al., who also identified increased mitophagy in CerS2 overexpressing human cardiomyocytes.

*MFN1* and *MFN2* were significantly reduced in FLAG-CerS2, supporting the microscope data and the findings of Chen et al. [27]. Pre-treatment with MT and FuB improved reduced *MFN2* expression but had no effects on *MFN1*. This indicates that the decrease of *MFN1* is not associated with ceramide levels but with CerS2 protein levels due to the CerS non-specificity of FuB. FuB pre-treatment further reduced *MFN1* expression in FLAG-CerS2. This paradox result could be explained by the varying transfection efficiency but further emphasizes the non-beneficial effects of FuB on *MFN1*. The respective Dox data also support this association. Here too, a significant reduction in *MFN1* was observed, which was unaffected by FuB-caused ceramide reduction. The knockdown of CerS2 and subsequent Dox treatment led to the increase in previously observed *MFN1* reduction. The knockdown data show that the expression of *MFN1* is not regulated by ceramide levels but directly by the CerS2 protein level. Further research is necessary to elucidate the detailed interconnection between CerS2 and *MFN1*.

The *MFN2* expression was unaffected by any of the used pre-treatments or the knockdown of CerS2, followed by Dox incubation. These results are contradictory to previous findings [28]. We hypothesize that the observed upregulation of *MFN2* could be a compensatory mechanism by the human fibroblasts. This potential counter mechanism was also observed in HL-1 cells [25] but still needs further investigations. FuB returned previously reduced *MFN2* expression to baseline levels in FLAG-CerS2 and suggests that the *MFN2* increasing effects of Dox superimpose the beneficial ceramide reducing effects of FuB.

*Mff* [29] and *FIS1* [30] were unchanged in Dox-treated fibroblasts, where an increase might have been expected. This could hint that the detected mitochondrial fission happens within the used incubation time of 24 h and that *Mff* and *FIS1* might be upregulated within this period and returned to a normal level afterward. The measurement of *Mff* and *FIS1* at an earlier time point could clarify the observed effects Preincubation with MT or FuB reduced the mRNA level of both genes, which was also detectable in FuB pre-treated FLAG-CerS2. The knockdown data concur and support our findings. *Mff* and *FIS1* were significantly reduced in knockdown cells followed by Dox treatment, showing the advantageous effects of ceramide reduction in Dox-mediated cell toxicity.

The reduction in mitochondrial ROS, either primary by MT or as a secondary effect of FuB, improves mitochondrial structure more by the downregulation of fission-related genes *Mff* and *FIS1* instead of the upregulation of the fusion-related genes (*MFN1, MFN2,* and *OPA1*). The fragmented mitochondrial structure also decreased mitochondrial function. Seahorse analysis revealed a significant reduction of OCR and, therefore, of mitochondrial ATP production in Dox and FLAG-CerS2. Interestingly, even though both inhibitors showed reduced inflammation and improved mitochondrial structure, no mitochondrial function improvement in Dox-treated cells was detected. This indicates that the detrimental effects of 0.7 µM Dox are too strong to be improved by ceramide reduction alone. Coherent to the impairment of mitochondrial homeostasis, is the induction of apoptosis. Dox and FLAG-CerS2 induce apoptosis by translocation of Cytochrome c and Casp9 activation. This activation is reduced by the preincubation of MT and FuB. Furthermore, siCerS2 also improves cell vitality by reduced Casp9 activation when compared to only Dox treated cells.

Fibrosis is a regenerative response to a pathogenic stimulus that can become maladaptive and result in a loss-of-function of the damaged tissue [31]. Our data have shown an increase in *MMP8* [32], *MMP9* [33], and *MMP14* [34] expression. Furthermore, the mRNA expression of the fibrosis marker *TGF-ß* [35] and *ACTA2* [36] were also increased. Additionally, the upregulation of *TIMP1*, *TIMP2* in Dox treated cells also indicates fibrosis progression [37]. Again, the reduction in ceramides also reduced fibrosis related gene expression except for *ACTA2*. The reduction of CerS2 with siRNA produced similar results to FuB pre-treatment. FLAG-CerS2 mainly affected *MMP8* and *MMP9* expression. Both genes already appeared at a later cT cycle in the qPCR in the control, and therefore they were no longer detectable after preincubation with FuB. The other measured genes only showed an increased tendency, which could be explained by varying transfection efficiency. CerS2 overexpression did not affect TIMP1 and TIMP2 expression and remained unchanged under FuB, while FuB reduced TIMP2 expression under Dox. This could also indicate that the mRNA expression is not directly linked to ceramides but to other CerS than CerS2. Still, the reduction in ceramides reduce fibrosis progression in Dox treated fibroblasts.

Our results implicate a strongly contributed connection between increased ceramides and the detrimental effects of Dox. Even so, some of our results were contrary to our expectations. An example is the mRNA expression of *MFN1* in FLAG-CerS2. Here, the overexpression led to a significant decrease in the gene, which was further enhanced upon the pre-treatment with FuB, where an increase in *MFN1* would have been expected. A single or combination of reasons could explain such paradox results. Variations of transfection efficiency or too low concentrations or incubation times of either FuB or MT could be an example for the observed contrary or not strong enough effects. Further modifications and improvements could explain the observed effects.

## 4. Material and Methods

### 4.1. CerS2 Overexpression and CerS2 Silencing

The original unedited p3xFLAG-CMV7 vector (FLAG-TC) and the p3xFLAG-CerS2 overexpression plasmid were designed and acquired from GENEWIZ (South Plainfield, NJ, USA). The cells were transfected with Lipofectamine 3000 (Thermo Fisher, Waltham, MA, USA).

The siRNA control, siCerS2, and RNAi Max were acquired from Thermo Fisher (Waltham, MA, USA) and used according to manufacturer’s protocol. Cells were transfected for 24 h in OptiMEM followed by 48 h in cell culture medium. Afterward, siRNA control was incubated in growth medium with solvent co and siCerS2 with 0.7 µM Dox for further 24 h.

### 4.2. Cell Culture

Human foreskin fibroblasts (hFSF) were acquired from ATCC (SCRC-1041^TM^) Manassas, Virginia, USA. The cells were cultured in DMEM (Thermo Fisher, Waltham, MA, USA) with 15% heat-inactivated fetal bovine serum (Merck, Darmstadt, Germany), 1% non-essential amino acids (Merck, Darmstadt, Germany), 1% L- glutamine (Merck), 1% sodium pyruvate (Merck, Darmstadt, Germany) and 0.5% Pen/Strep (Merck, Darmstadt, Germany). Dox and MT were acquired from Merck (Darmstadt, Germany). FuB was acquired from Cayman chemical (Ann Arbor, MI, USA).

Solvent control (solvent co) for Dox and MT was H_2_O. Solvent co for FuB was DMSO. Results were normalized to solvent co (H_2_O) or otherwise indicated. Transfection results were normalized to FLAG-TC, siRNA control, or otherwise indicated.

### 4.3. RNA Isolation and mRNA Expression Level Validation

RNA isolation was conducted with NucleoSpin RNA (Macherey Nagel, Düren, Germany) according to protocol. The RevertAid First Strand cDNA Synthesis Kit (Thermo Fisher, Waltham, MA, USA) was used for cDNA synthesis, also according to manufacturer’s protocol. Gene expression was determined with qPCR. Inflammatory cytokine expression of tumor necrosis factor-alpha (*TNFα*), interleukin- 6 (*IL-6*), and interleukin-1β (*IL-1β*) was measured. For evaluation of ROS homeostasis, the ROS scavenger superoxide dismutase 2 (*SOD2*), glutathione peroxidase 1 (*GPx1*), catalase (*Cat*), and the ROS inducer NADPH oxidase 2 (*NOX2*) were assessed. For metabolic associated genes diacylglycerol O-acyltransferase 1 (*DGAT1*), diacylglycerol O-acyltransferase 2 (*DGAT2*), adipocyte- triglyceride- lipase (*ATGL*), CD36 und carnitine palmitoyltransferase 1 beta (*CPT1B*), pyruvate dehydrogenase kinase isoenzyme 4 (*PDK4*), and glutamate dehydrogenase (*GDH*) were quantified. Tested mitochondria fusion and fission genes were mitofusin 1 (*MFN1*), mitofusin 2 (*MFN2*), dynamin-like 120 kDa protein (*OPA1*), dynamin-1-like protein (*DRP1*), mitochondria fission factor (*Mff*), and mitochondrial fission 1 protein (*FIS1*). Fibrosis-related genes such as matrix metalloproteinase (*MMP*), Tissue inhibitor of metalloproteinase 1 and 2 (*TIMP1, TIMP2*), transforming growth factor β (*TGF-β*), alpha smooth muscle actin (ACTA2) was also probed (Supplementary Table S1).

### 4.4. Protein Isolation and Protein Expression Level Detection

Cells were lysed in RIPA lysis buffer (50 mM TRIS pH 7.4, 150 mM NaCl, 1% Triton-X-100, 0.1% SDS, 0.5% Deoxycholate). Protein concentration was determined with Bradford assay. Specific protein expression was assessed with SDS-PAGE and WB. Vinculin, FLAG, CerS2, and secondary antibodies were acquired from Abcam (Cambridge, UK). Caspase 9 antibodies were acquired from Cell Signaling (Danvers, MA, USA).

### 4.5. Ceramide Content

Lipids were extracted following Folch extraction [38] under acidified conditions. Three µM d18:1/15:0 ceramide in methanol as an internal standard were added to the cells. 300 µL HCl, 1 mL methanol, and 2 mL chloroform were added, and the samples were vortexed for 10 min at full speed and centrifuged at 1900× *g* for 3 min. Lower organic phase containing ceramides was transferred to a new glass tube. Organic phase was evaporated to dryness for 45 min at 50 °C and reconstituted in 100 µL 1:4 chloroform: methanol. For ceramide detection, the Prominence 20A series HPLC (Shimadzu, Duisburg, Germany) coupled to the 2000QTrap triple-quadrupole mass spectrometer (Sciex, Darmstadt, Germany) equipped with an APCI source operating in positive mode was used. Standard solutions were acquired from Avanti Polar Lipids Inc. (Alabaster, AL, USA) and added for ceramide identification. The analytical results were quantified with Analyst 1.6.2 (Sciex, Darmstadt, Germany) based on external standard curves.

### 4.6. Ceramide Detection with FACS

Ceramide antibody (Enzo, Famingdale, NY, USA) staining was conducted with BD Cytofix/Cytoperm Kit (Franklin Lakes, NJ, USA). Primary antibody (1:100) was used for 1 h at room temperature. The secondary antibody was used at 1:200 (abcam) dilution for 30 min. Fluorescence was detected with FACS (FACSCalibur, BD Biosciences, Franklin Lakes, NJ, USA) at 633 nm. Quantification was achieved with FlowJo software.

### 4.7. Immunohistochemistry

Fixation of cells was achieved with Methanol: Acetone (7:3). Primary anti-CerS2 antibody (Abcam, Cambridge, UK) and secondary antibody (Abcam, Cambridge, UK) were used in a 1:200 dilution. Primary Anti-ACTA2 antibody (Agilent Dako, Santa Clara, CA, USA) was used in a 1:50 dilution. Nucleus was stained with Hoechst (Life Technologies, Carlsbad, CA, USA) in a 1:5000 dilution. Fluorescence detection was accomplished with confocal laser scanning microscopy (cLSM, Zeiss).

Mitochondrial structure illustration was conducted with MitoTracker (Thermo Fisher, Waltham, MA, USA) diluted 1:2000. Nuclei were stained with 1:5000 diluted Hoechst. Fluorescence detection was accomplished with cLSM.

### 4.8. Electron Microscopy

Cells were treated with freshly prepared fixation buffer (2.5% Glutaraldehyde, 20 mM HEPES pH 4, 135 mM NaCl, 5 mM KCl, 1 mM MgSO_4_, 1.5 mM CaCl_2_) for 1 h at room temperature. The cells were washed and resuspended with 0.1 M Cacodylate- buffer pH 7.4. After post-fixation in osmium tetroxide for 1 h, dehydration in ascending ethanol series with post-staining in uranyl acetate was performed. Afterward, the samples were embedded in epoxy resin (Araldite) and sectioned using a Leica Ultracut E (Leica, Wetzlar, Germany). Based on the examination of semi-thin sections, regions of interest of about 500 µm × 500 µm in size were selected and trimmed. Finally, ultrathin sections were mounted on filmed Cu grids, post-stained with lead citrate, and studied in a transmission electron microscope (EM 900, Zeiss, Oberkochen, Germany) at 80 kV and magnifications of 3000× to 20,000×.

### 4.9. Mitochondrial ROS Measurement with MitoSOX

The cells were incubated in a serum-free medium for 1 h directly before ROS measurement. MitoSOX (Invitrogen, Waltham, MA, USA) was added in a 1:1000 dilution and incubated for 20 min. Fluorescence detection was accomplished with cLSM.

### 4.10. Seahorse Analysis

Mitochondrial ATP production measurement was conducted with the live-cell metabolic assay platform seahorse analyzer XF^e^ 96 and the Mito Stress Kit (Agilent Technologies, Santa Clara, CA, USA). Oligomycin (2 µM), FCCP (0.3 µM), and Rotenone/Antimycin (0.5 µM) were applied according to company advisement.

### 4.11. Collagen Measurement in Cell Supernatant

Collagen concentration was determined with the Soluble Collagen Assay Sircol (biocolor, Carrickfergus, UK). Cells were cultivated in a 96 well plate with 250 µL medium and treated as indicated. Samples were processed according to protocol. Absorption was measured at 555 nm with the TECAN Infinite M1000 Pro.

### 4.12. Statistical Analysis

Statistical analysis was performed with SigmaPlot 14.0. Data are depicted as mean values ± standard deviation as our standard way of data presentation and SD is a better representation of the data distribution and normalized to the respective control. Significance was calculated with the student’s *t*-test (*p* ≤ 0.05). The graphs were designed with GraphPad Prism 8.

## Figures and Tables

**Table 1:** ceramide levels in Dox treated and FuB pretreated fibroblasts.

Ceramide	Solvent co	0.7 µM Dox	FuB + Dox
Total Ceramides	1.0 ± 0.07	1.39 ± 0.01	0.70 ± 0.11
(C14–C24:1)		(*p* = 0.002 *)	(*p* < 0.001 *)
			(*p* < 0.001 #)
C14	1.0 ± 0.04	1.20 ± 0.01	0.21 ± 0.39
		(*p* = 0.003 *)	(*p* < 0.001 *)
			(*p* < 0.001 #)
C16	1.0 ± 0.05	1.28 ± 0.02	1.03 ± 0.09
		(*p* = 0.002 *)	(*p* = 0.67)
			(*p* = 0.001 #)
C18	1.0 ± 0.07	1.29 ± 0.02	1.04 ± 0.09
		(*p* = 0.006 *)	(*p* < 0.001 *)
			(*p* < 0.001 #)
C20	1.0 ± 0.03	1.24 ± 0.02	0.12 ± 0.41
		(*p* < 0.001 *)	(*p* < 0.001 *)
			(*p* < 0.001 #)
C22	1.0 ± 0.12	1.54 ± 0.03	0.72 ± 0.09
		(*p* = 0.004 *)	(*p* < 0.001 *)
			(*p* < 0.001 #)
C22:1	1.0 ± 0.08	1.45 ± 0.03	0.35 ± 0.13
		(*p* = 0.002 *)	(*p* < 0.001 *)
			(*p* < 0.001 #)
C24	1.0 ± 0.23	1.64 ± 0.02	0.69 ± 0.17
		(*p* = 0.02 *)	(*p* = 0.02 *)
			(*p* < 0.001 #)
C24:1	1.0 ± 0.03	1.33 ± 0.02	0.52 ± 0.14
		(*p* < 0.001 *)	(*p* < 0.001 *)
			(*p* < 0.001 #)

Depicted are mean values and standard deviation of three independent measurements. Significance was calculated with a *t*-test (*p* ≤ 0.05). * = significant to solvent co. # = significant to 0.7 µM Dox.). solvent co = solvent control. Dox = Doxorubicin, FuB = Fumonisin B1.

**Table 2:** Mitochondria fusion and fission related mRNA expression.

Gene	Solvent Co	0.7 µM Dox	MT + Dox	FuB + Dox
*MFN1*	1.0 ± 0.09	0.80 ± 0.14	0.77 ± 0.17	0.73 ± 0.22
		(*p* = 0.007 *)	(*p* = 0.004 *)	(*p* = 0.002 *)
*MFN2*	1.0 ± 0.28	6.79 ± 0.40	6.65 ± 0.20	5.91 ± 0.23
		(*p* < 0.001 *)	(*p* < 0.001 *)	(*p* < 0.001 *)
*OPA1*	1.0 ± 0.16	0.66 ± 0.18	0.49 ± 0.10	0.51 ± 0.26
		(*p* = 0.002 *)	(*p* < 0.001 *)	(*p* < 0.001 *)
			(*p* = 0.03 #)	
*DRP1*	1.0 ± 0.19	0.74 ± 0.21	0.65 ± 0.21	0.64 ± 0.12
		(*p* = 0.03 *)	(*p* = 0.004 *)	(*p* = 0.002 *)
*Mff*	1.0 ± 0.18	0.90 ± 0.16	0.77 ± 0.15	0.63 ± 0.29
			(*p* = 0.02 *)	(*p* = 0.002 *)
				(*p* = 0.03 #)
*FIS1*	1.0 ± 0.15	0.89 ± 0.14	0.77 ± 0.10	0.68 ± 0.10
			(*p* = 0.01 *)	(*p* < 0.001 *)
				(*p* = 0.02 #)

Depicted are mean values and standard deviation of minimum 3 independent measurements. Significance was calculated with a *t*-test (*p* ≤ 0.05). * = significant to solvent co. # = significant to 0.7 µM Dox.

**Table 3:** Metabolism related mRNA expression.

Gene	Solvent Co	0.7 µM Dox	MT + Dox	FuB + Dox
*DGAT1*	1.0 ± 0.30	2.65 ± 0.25	2.86 ± 0.28	3.24 ± 0.47
		(*p* < 0.001 *)	(*p* < 0.001 *)	(*p* = 0.006 *)
*DGAT2*	1.0 ± 0.29	1.95 ± 0.22	1.44 ± 0.50	1.37 ± 0.24
		(*p* = 0.06 *)		
*ATGL*	1.0 ± 0.38	3.02 ± 0.18	3.64 ± 0.22	2.26 ± 0.33
		(*p* < 0.001 *)	(*p* < 0.001 *)	(*p* = 0.01 *)
*CD36*	1.0 ± 0.58	4.02 ± 0.53	4.83 ± 0.24	3.35 ± 0.54
		(*p* < 0.001 *)	(*p* < 0.001 *)	(*p* = 0.002 *)
*CPT1B*	1.0 ± 0.40	0.28 ± 0.38	0.20 ± 0.58	0.19 ± 0.82
		(*p* = 0.005 *)	(*p* = 0.006 *)	(*p* = 0.002 *)
*PDK4*	1.0 ± 0.25	0.55 ± 0.35	0.30 ± 0.25	0.46 ± 0.40
		(*p* = 0.01 *)	(*p* = 0.002 *)	(*p* = 0.008 *)
			(*p* = 0.05 #)	
*GDH*	1.0 ± 0.15	0.89 ± 0.35	1.20 ± 0.20	0.84 ± 0.27

Depicted are mean values and standard deviation of minimum 3 independent measurements. Significance was calculated with a *t*-test (*p* ≤ 0.05). * = significant to solvent co. # = significant to 0.7 µM Dox.

**Table 4:** Fibrosis related mRNA expression.

Gene	Solvent Co	0.7 µM Dox	MT + Dox	FuB + Dox
*MMP8*	1.0 ± 0.32	3.65 ± 0.20	2.91 ± 0.49	1.46 ± 0.32
		(*p* < 0.001 *)	(*p* = 0.03 *)	(*p* = 0.001 #)
*MMP9*	1.0 ± 0.25	3.31 ± 0.52	2.72 ± 0.52	1.69 ± 0.53
		(*p* = 0.01 *)	(*p* = 0.03 *)	
*MMP14*	1.0 ± 0.22	1.53 ± 0.24	1.20 ± 0.30	1.14 ± 0.41
		(*p* = 0.01 *)		
*TIMP1*	1.0 ± 0.18	1.63 ± 0.14	2.89 ± 0.33	1.86 ± 0.21
		(*p* = 0.005 *)	(*p* = 0.005 *)	(*p* = 0.006 *)
*TIMP2*	1.0 ± 0.04	1.66 ± 0.20	1.38 ± 0.29	1.32 ± 0.24
		(*p* = 0.01 *)		
*TGF-β*	1.0 ± 0.14	2.26 ± 0.23	1.45 ± 0.37	1.24 ± 0.50
		(*p* = 0.01 *)		
*ACTA2*	1.0 ± 0.26	2.00 ± 0.32	2.12 ± 0.37	2.00 ± 0.28
		(*p* = 0.009 *)	(*p* = 0.02 *)	(*p* = 0.005 *)

Depicted are mean values and standard deviation of minimum three independent measurements. Significance was calculated with a *t*-test (*p* ≤ 0.05). * = significant to solvent co. # = significant to 0.7 µM Dox.

**Table 5:** Ceramide levels in transfected fibroblasts.

Ceramide Species	FLAG-TC	FLAG-CerS2	*p* Value
Total Ceramides (C14–C24:1)	1.0 ± 0.29	2.12 ± 0.08	0.009
C14	1.0 ± 0.36	1.32 ± 0.09	0.30
C16	1.0 ± 0.82	2.83 ± 0.09	0.04
C18	1.0 ± 0.16	2.27 ± 0.11	0.004
C20	1.0 ± 0.11	1.28 ± 0.12	0.10
C22	1.0 ± 0.19	2.90 ± 0.11	0.002
C22:1	1.0 ± 0.18	0.92 ± 0.06	0.56
C24	1.0 ± 0.29	2.89 ± 0.15	0.006
C24:1	1.0 ± 0.24	1.51 ± 0.02	0.04

Depicted are mean values and standard deviation of minimum three independent measurements. Significance was calculated with a *t*-test (*p* ≤ 0.05).

**Table 6:** ceramide levels in FuB pre-treated and transfected fibroblasts.

Ceramide Species	DMSO+ FLAG-TC	FuB + FLAG-CerS2	*p* Value
Total Ceramides (C14–C24:1)	1.0 ± 0.34	0.85 ± 0.23	0.42
C14	1.0 ± 0.20	0.56 ± 0.25	0.002
C16	1.0 ± 0.32	0.90 ± 0.26	0.59
C18	1.0 ± 0.18	0.97 ± 0.16	0.77
C20	1.0 ± 0.13	1.11 ± 0.35	0.57
C22	1.0 ± 0.31	0.90 ± 0.26	0.55
C22:1	1.0 ± 0.05	1.03 ± 0.14	0.66
C24	1.0 ± 0.47	0.64 ± 0.35	0.16
C24:1	1.0 ± 0.40	0.88 ± 0.22	0.56

Depicted are mean values and standard deviation of minimum three independent measurements. Significance was calculated with a *t*-test (*p* ≤ 0.05).

**Table 7:** Mitochondria fusion and fission related mRNA expression in transfected Fibroblasts.

Gene	FLAG-TC	FLAG-CerS2	MT + FLAG-CerS2	FuB + FLAG-CerS2
*MFN1*	1.0 ± 0.01	0.86 ± 0.07	0.72 ± 0.25	0.30 ± 0.21
		(*p* = 0.04 *)		(*p* < 0.001 *)
				(*p* < 0.001 #)
*MFN2*	1.0 ± 0.11	0.68 ± 0.10	1.03 ± 0.11	1.14 ± 0.31
		(*p* = 0.02 *)	(*p* = 0.02 #)	
*OPA1*	1.0 ± 0.08	0.82 ± 0.13	0.63 ± 0.14	0.51 ± 0.15
			(*p* = 0.01 *)	(*p* = 0.003 *)
				(*p* = 0.03 #)
*DRP1*	1.0 ± 0.05	0.89 ± 0.05	0.74 ± 0.18	0.44 ± 0.22
				(*p* = 0.002 *)
				(*p* = 0.004 #)
*Mff*	1.0 ± 0.06	0.79 ± 0.06	0.78 ± 0.16	0.72 ± 0.07
		(*p* = 0.02)		(*p* = 0.006 *)
*FIS1*	1.0 ± 0.08	0.89 ± 0.10	0.71 ± 0.13	0.63 ± 0.06
			(*p* = 0.03 *)	(*p* = 0.003 *)
				(*p* = 0.02 #)

Depicted are mean values and standard deviation of minimum three independent measurements. Significance was calculated with a *t*-test (*p* ≤ 0.05). * = significant to FLAG-TC. # = significant to FLAG-CerS2.

**Table 8:** Metabolism related mRNA expression in transfected fibroblasts.

Gene	FLAG-TC	FLAG-CerS2	MT + FLAG-CerS2	FuB + FLAG-CerS2
*DGAT1*	1.0 ± 0.21	1.03 ± 0.19	1.23 ± 0.21	0.67 ± 0.03
				(*p* = 0.03 #)
*DGAT2*	1.0 ± 0.21	0.66 ± 0.31	0.45 ± 0.41	0.40 ± 0.44
		(*p* = 0.03 *)	(*p* = 0.01 *)	(*p* = 0.007 *)
*ATGL*	1.0 ± 0.24	1.20 ± 0.37	0.80 ± 0.16	0.79 ± 0.05
*CD36*	1.0 ± 0.85	3.61 ± 0.26	4.57 ± 0.34	1.25 ± 0.53
		(*p* < 0.001 *)	(*p* = 0.002 *)	(*p* = 0.01 #)
*CPT1B*	1.0 ± 0.38	0.42 ± 0.65	0.34 ± 0.35	0.24 ± 0.67
		(*p* = 0.02 *)	(*p* = 0.05 *)	(*p* = 0.03 *)
*PDK4*	1.0 ± 0.38	0.86 ± 0.23	0.64 ± 0.26	0.85 ± 0.56
*GDH*	1.0 ± 0.24	0.92 ± 0.23	0.85 ± 0.12	0.72 ± 0.16

Depicted are mean values and standard deviation of minimum three independent measurements. Significance was calculated with a *t*-test (*p* ≤ 0.05). * = significant to FLAG-TC. # = significant to FLAG-CerS2.

**Table 9:** Fibrosis related mRNA expression in transfected fibroblasts.

Gene	FLAG-TC	FLAG-CerS2	MT + FLAG-CerS2	FuB + FLAG-CerS2
*MMP8*	1.0 ± 0.17	2.55 ± 0.53	1.49 ± 0.36	n.d.
		(*p* = 0.03 *)		
*MMP9*	1.0 ± 0.36	1.45 ± 0.69	1.25 ± 0.41	n.d.
*MMP14*	1.0 ± 0.33	0.91 ± 0.21	1.04 ± 0.10	7.17 ± 0.16 * 10^−5^
				(*p* = 0.004 *)
				(*p* < 0.001 #)
*TIMP1*	1.0 ± 0.40	1.09 ± 0.52	1.12 ± 0.41	1.29 ± 0.07
*TIMP2*	1.0 ± 0.09	0.93 ± 0.19	1.06 ± 0.07	0.96 ± 0.14
*TGF-β*	1.0 ± 0.18	1.23 ± 0.36	1.32 ± 0.39	1.72 ± 0.19
				(*p* = 0.03 *)
*ACTA2*	1.0 ± 0.23	0.72 ± 0.14	1.25 ± 0.09	1.19 ± 0.13
			(*p* = 0.007 #)	(*p* = 0.02 #)

Depicted are mean values and standard deviation of minimum three independent measurements. Significance was calculated with a *t*-test (*p* ≤ 0.05). * = significant to FLAG-TC. # = significant to FLAG-CerS2. n.d. = not detectable.

## Data Availability

All data, tables and figures in this manuscript are original. The data presented in this study are available in the article or Supplementary Material.

## Supplementary Materials

The following are available online at https://www.mdpi.com/article/10.3390/ijms222111852/s1.
