# Supplementary figures and images for: Long-Chain and Very Long-Chain Ceramides Mediate Doxorubicin-Induced Toxicity and Fibrosis
Source: Int J Mol Sci. 2021 Nov 1;22(21):11852. doi: 10.3390/ijms222111852 (PMC8584314; doi:10.3390/ijms222111852)

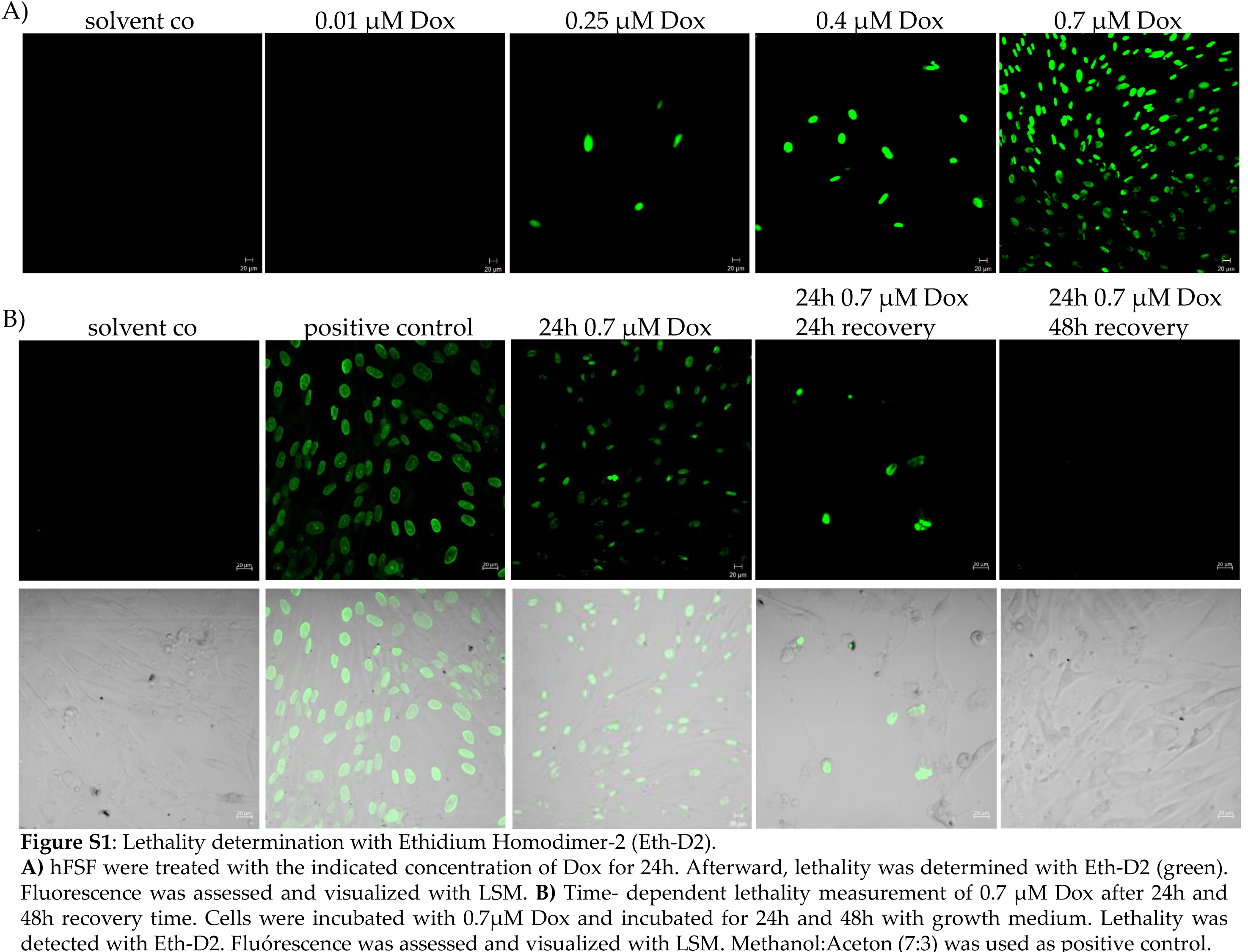

Supplement: Supplementary file 1 [file ijms-22-11852-s001.zip › Supplement Figure S1_rev.tif]

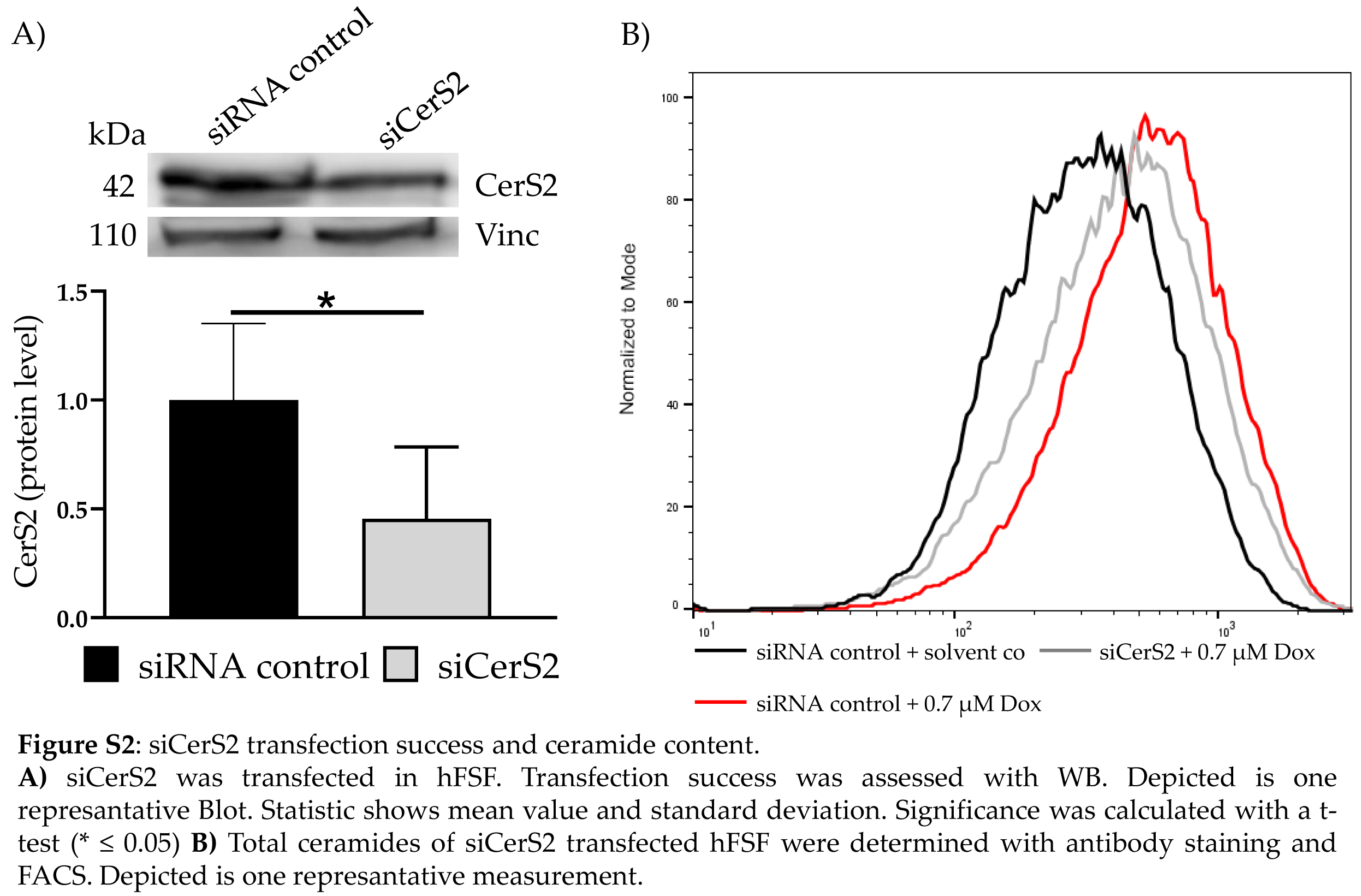

Supplement: Supplementary file 1 [file ijms-22-11852-s001.zip › Supplement Figure S2_rev.tif]

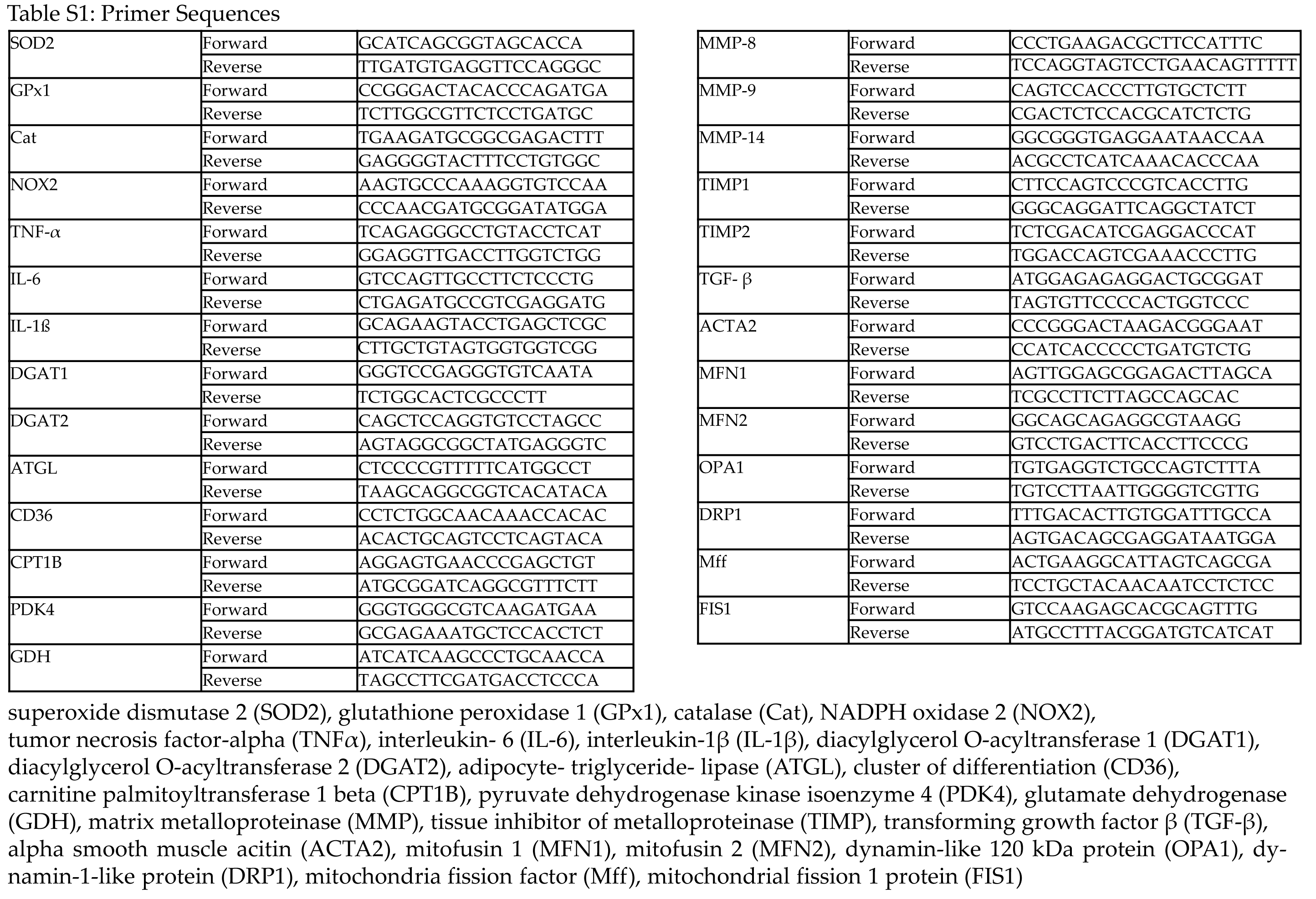

Supplement: Supplementary file 1 [file ijms-22-11852-s001.zip › Supplement table S1_rev.tif]
